# A Comprehensive and Unified Approach to Web Service Trust Evaluation Based on Uncertainty Methodology

**DOI:** 10.3390/e24020243

**Published:** 2022-02-05

**Authors:** Junwei Zhang, Deyu Li

**Affiliations:** 1 School of Computer and Information Technology, Shanxi University, Taiyuan 030006, China; junwei_zhang@sxmu.edu.cn; 2 School of Management, Shanxi Medical University, Taiyuan 030001, China

**Keywords:** service composition, service selection, trust bootstrap, trust derivation, uncertainty, web services

## Abstract

Web services have the advantage of being able to generate new value-added services based on existing services. To effectively compose Web services, the composition process necessitates that the services that will participate in a given composite service are more trustworthy than those that provide similar functionality. The trust mechanism appears to be a promising way for determining service selection and composition. Existing trust evaluation approaches do not take into account customer expectations. Based on fuzzy set theory and probability theory, this work proposes a unique Web service trust evaluation approach that is notable for its ability to provide personalized service selection based on customer expectations and preferences. The proposed approach defines trust as a fuzzy notion that is related to prior experiences and ratings, and expresses trust in two different forms. This work mainly solves two key issues in Web service trust architectures, bootstrapping trust for the newcomer services and deriving trust for composite services. The proposed approach combines the solutions to numerous issues in a natural way. The case study and approaches comparison demonstrate that the proposed approach is feasible.

## 1. Introduction

Currently, many organizations deploy various applications for business management. Rather than developing new solutions in-house, these applications are frequently constructed by integrating components produced by outside firms [1,2,3]. Web services are self-contained application components created on many platforms and advertised, hosted, and accessed via the Internet [4,5,6]. An elementary Web service is a network-accessible process that performs a defined task. When two or more elementary Web services are combined, a new Web service is created that performs a complex composite task that no single elementary service can accomplish alone [7,8,9]. In this case, the new Web service that contains the elementary Web services is called the composite service, and these elementary Web services are called the component services. The process of creating composite services can be performed iteratively, with one composite service serving as a component of a more complicated composite service. The true power of Web services, as demonstrated above, is their ability to be dynamically integrated into composite services, performing new tasks and conducting new business transactions [10,11,12].

To create a successful composite service, the correct component services need to be selected [2]. In the sector of Web services, how to select desirable Web services and composite appropriate Web services has been a major issue. Both for direct interactions and for specifying composite services, service selection is critical. The rapid growth of the Internet has resulted in a massive increase in the number of Web services, posing a challenge to consumers’ ability to quickly and precisely locate their desirable services [13,14,15]. Furthermore, an increasing number of Web services from diverse service providers provide the same functionality [16]. Businesses today must comprehend not just what a Web service can do but also how well the Web service can do it. How to select the desired component services and recommend the satisfactory composite services are very important issues [17]. The non-functional characteristics of Web services, such as QoS (Quality of Service) and trust, are very essential criteria when selecting and composing the desirable Web services [18,19,20,21].

The QoS determines the performance of a Web service [22], so the QoS is regarded as the most important non-functional criterion for service selection and composition [23,24,25]. However, the QoS that a given Web service will supply is not predictable and foreseeable due to dynamic changes in network performance [3,8,17,26,27,28]. Furthermore, various customers may be concerned with different QoS attributes, and different customers may assign different priority to the same QoS attributes [12,29,30]. To replace QoS-aware service selection and composition procedures, the attention has recently shifted to social techniques based on trust or reputation. The trust mechanism provides a smart and accurate solution to the issue of service selection and composition [2,31].

Trust in a Web service represents a certain level of confidence that the service will perform as expected [2]. Trust should be applicable to both elementary and composite services. Trust is based on a consumer’s past interactions with or observations of a Web service, either directly with the customer or as reported by other customers. Each customer in a community can function as a rater, giving a service a rating based on his/her interactions with it. Individuals’ ratings on the quality of a service are reflected in trust, which is customized and subjective. The higher a service’s quality, the more trust the consumer has in it. Trust can be used to assess the overall quality of a service [32]. A trust model for Web services integrates past experiences or interactions to generate trust, allowing customers to identify good from bad services based on customers’ ratings, and assisting customers in forecasting and selecting the best quality services [33,34].

Because trust can only exist in a risky and uncertain environment, where certain parts of the application are not within our control, trust is inherently uncertain. Our previous work defined trust in Web services using a mathematical model based on fuzzy theory and probability theory, and presented a customer-centric trust evaluation model for personalized service selection [35]. However, our previous work did not consider bootstrapping trust for the newcomer services and deriving trust for the composite services, which are two key issues in Web service trust architectures. This work will improve the previous work to form a complete Web service trust evaluation approach.

This work combines the solutions to numerous issues to develop a comprehensive and unified Web service trust evaluation approach. A motivation example is discussed in Section 2. The present research on bootstrapping trust for the newcomer services and deriving trust for the composite services are investigated in Section 3. The integrated trust evaluation approach is described in Section 4. Through a case study, Section 5 describes the proposed approach’s practical applicability. This work compares the proposed approach to existing approaches in Section 6. Section 7 of the work finishes with an overview of the vast potentials of such an approach.

## 2. Motivation

First and foremost, a motivating case is offered to emphasize this work’s motivation.

Service-oriented architecture (SOA) systems have capabilities to dynamically compose Web services [6,36], including the following:

- Service tracking to detect all available services and their rating information;
- Service planning to design a composite service’s execution process;
- Service selection to determine the optimum service for each task node in a composite service process;
- Service composition to generate a new service that performs the expected complex task by compositing the selected component services;
- Service execution to perform the expected complex task according to the optimal composed plan.

**Figure 1:**
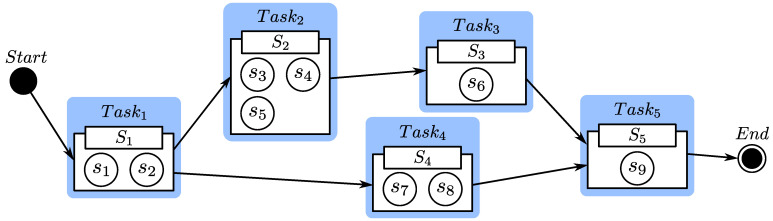
An example of service selection and service composition.

A service workflow, i.e., a composite service, includes a set of tasks [37]. According to service planning, there may be more than one way to build a composite service [36]. Figure 1 depicts a composite service that can take one of two paths: $Task_{1}$, $Task_{2}$, $Task_{3}$, $Task_{5}$ or $Task_{1}$, $Task_{4}$, $Task_{5}$. Each task $Task_{i}$ may be performed by any elementary service in service class $S_{i}$. The rating information of each elementary service is shown in Table 1.

Assume that Alice and Bob both need to select desired services in order to build the composite service. Table 1 shows that Alice and Bob are faced with the following three service selection scenarios:

1. Alice and Bob have used and rated all of the candidate services (for example, in service class $S_{1}$). They can make decisions directly based on their own ratings. Of course, they can also make decisions based on ratings from previous customers who have used these services.
2. Alice and Bob have never used any of the candidate services and have no knowledge of how well they perform (for example, in service class $S_{2}$). Only ratings submitted by other consumers who have used these services can help them make decisions.
3. Alice and Bob have used some of the candidate services, but they have no knowledge of the performance of others (for example, in the service class $S_{4}$). They can make decisions based on ratings submitted by other consumers who have used these services, similar to the second scenario.

In this motivating example, there are three issues that should be noted. The first issue is to bootstrap trust for new Web services. $s_{3}$ is a new Web service in service class $S_{2}$. No consumer has interacted with it, and no rating exists of its past behavior. Consequently, consumers are unable to evaluate its trustworthiness. A mechanism for assigning trust to new services must be specified so that they can compete for market share with existing services. The second issue is to reduce the influence of subjectivity and contingency when the ratings are few. In service class $S_{4}$, there are only three ratings on $s_{7}$, while there are 1540 ratings on $s_{8}$. These three ratings cannot completely and effectively reflect the performance of $s_{7}$, so consumers need to reduce the decision-making risk caused by the subjective and contingency when ratings on services are few. This issue is closely related to the first issue. The third issue is deriving trust for composite services. The composite service has two alternative paths, so customers need to choose one to execute. Choosing the option to implement the composite service is actually a decision to implement a more trustworthy composite service. Another mechanism must be defined to derive trust for composite services, allowing customers to select them as elementary services.

Our previous work has modeled the uncertainty of trust and implemented personalized service selection. This work will improve our previous work and address the following issues:

1. How do new services without past experience and customer ratings take part in the competition;
2. How to derive the trust value of a composite service according to the trust values of its component services while ensuring that the resultant trust values have the same properties.

## 3. Related Work

Trust in Web services is a critical prerequisite for their widespread acceptance in an open and dynamic network environment [10,38]. To ensure Web service trust, a set of trust criteria and frameworks should be supplied. Academics have different perspectives on trust, so studies were based on distinct perspectives [39]. Some academics investigated policy-based Web service trust, while others investigated reputation-based Web service trust. Mahmud and Usman conducted a systematic literature review with the aim of identifying and classifying existing studies on trust establishment and estimation in services [40]. This work’s major goal is to investigate reputation-based trust in Web service, so here we mainly survey the relevant literature on reputation-based trust evaluation approaches for Web services. A reputation-based trust system can be used to identify suitable services or service providers based on past experiences or consumer ratings and gauge customer satisfaction with Web services [41].

Web services are typically represented and defined over three main architectures: single, composite, and communities [33], and this work deals with both single and composite architectures. Trust evaluation research in the single architecture focuses on bootstrapping trust, rating credibility, trust dynamics, and the processes of developing and upgrading trust or reputation. Our previous work has addressed issues other than bootstrapping trust.

Bootstrapping trust for a newcomer Web service means assigning an initial trust value for it [42], which is a key issue in Web service trust models [43,44]. Because historical information on newcomer Web services is unavailable, bootstrapping trust becomes a significant difficulty that most existing Web service trust models overlook [44,45]. Malik and Bouguettaya looked at a variety of ways for bootstrapping new Web service reputations equitably and properly, as well as their shortcomings [44]. Existing bootstrapping trust solutions fall into three main categories: default, punishment-based, and adaptive [46]. The default value-based approach assigns a default trust value to each newcomer service, while the punishment-based approach assigns a low trust value to newcomer services. Both these two approaches have drawbacks; if the initial trust value is high, existing services will be disadvantaged, while a low initial trust value will prevent newcomer services from competing. The adaptive bootstrapping trust approaches assign the initial trust value of a newcomer service by measuring the similarity between the newly deployed service and some existing services. The adaptive bootstrapping is the current resident bootstrapping trust solution. Nguyen et al. presented a trust bootstrapping approach with three auxiliary mechanisms, including inheritance, recommendation, and assurance mechanisms, which is distinguished by the fact that the bootstrapping mechanisms are combined with existing trust models to maximize the accuracy of the trust assessment process in the initial stage [45]. This approach is based on the characteristics and nature of the newcomer Web service itself, as well as the community, in order to apply appropriate bootstrapping mechanisms so that the assigned initial trust value is truly indicative of the newcomer’s capabilities. Yahyaoui and Zhioua introduced a new approach to bootstrap Web service trust by observing Web service interactions with customers over a certain time horizon [42]. The observation sequence is modeled as a Hidden Markov Model and matched with a predefined trust model to evaluate the behavior of such a Web service. The predefined trust model is a specification of the possible behavior of the Web service. Based on the matching results, an initial trust value is assigned to the Web service. Wahab and his colleagues suggested a peer-reviewed solution [46]. The trust bootstrapping mechanism is made up of a machine learning method for obtaining endorsements for newcomer services, a dishonesty-resistant endorsement aggregation technique, and a credibility updating mechanism for endorsers. Wu et al. presented a new technique to reputation bootstrapping in which artificial neural networks are utilized to learn correlations between existing service attributes and performance and then extend them to generate a provisional reputation when evaluating new and unknown services. The reputation bootstrapping also includes the reputations of previously published services from the same provider [47]. The above-mentioned bootstrapping trust methods are all adaptive methods, and the common shortcoming of these methods is that they require some priori information, but the bootstrapping trust problem is precisely caused by the lack of information.

The trust evaluation problem of composite services is a major focus of composite architecture research. It is important to involve trust in Web service composition when generating trustworthy composite Web services [48]. Yang et al. proposed a trust management system based on understanding and trust policies that are built on experience [49]. The trust of the participating Web services is assessed based on previous experiences, allowing the composite service’s trust to be determined based on the evaluation of each Web service and the composite service’s aggregation topology. Paradesi et al. conceptualized behavior-based trust of Web services with a mathematical model that matches many of the intuitions one has about trustworthy Web services [50]. The model allows modeling the uncertainty of people’s initial trust or distrust of Web services when there is no prior experience dealing with them. As the number of experiences with Web services increases, the level of certainty increases, although the proportion of positive experiences may remain fixed. Furthermore, when the same number of positive and negative experiences are gained, certainty is lowest. They also showed how the trust model for a single service may be used to derive trust for compositions. Yahyaoui suggested a trust-based game theoretical model with the goal of simulating the rivalry between services desiring to be assigned tasks and selecting the best candidate [51]. Web services utilize a Bayesian model to calculate a trust value for any other service willing to collaborate with them and then play a game to choose the best candidates. Kim et al. proposed a trust model that allows for the discovery and composition of services depending on their trustworthiness [52]. To determine the trustworthiness of services and service providers, the model relied on both direct and indirect consumer experience. Consumers can obtain a very trustworthy service that meets their quality and functional needs by using composing services. Gao et al. presented a formal service composition architecture for service selection [23]. They offered a subjective probability-theory-based trust evaluation approach for service composition plans, as well as a trust-oriented genetic algorithm to generate a near-optimal service composition plan with QoS limitations. Karimian et al. proposed a formulation for computing trust based on the transferable belief model using the basic concepts of belief combination and transferability [53]. Customer ratings of different interactions are combined to form a direct trust value. Trust in composite services is modeled over time using the generalized Bayes theorem in the transferable belief model, which takes into account the structure of each component in the service. Guo et al. proposed a trust-based service composition framework that uses a static program analysis approach to analyze the trust dependency between component services in a composite service. Based on this, the rating and trust dependency information is aggregated into a global trust calculation for the composite service [20]. It is important to note that a composite service can be used as a component of another more complex composite service. These current approaches to deriving trust for composite services lack the ability to generalize further.

Although there are already many approaches to serve bootstrapping trust for the newcomer services and deriving trust for the composite services, these existing trust evaluation approaches are limited in their scope of addressing issues. Their shortcomings are mainly as follows:

1. Existing trust evaluation approaches tend to focus only on one or some specific issues of trust and cannot cover the whole lifecycle and scope of trust in Web services.
2. Existing trust evaluation approaches often involve only customer preferences, and the subjective initiative of customers in the trustworthy representation is insufficient.
3. Bootstrapping trust for the newcomer services often requires additional information, but in fact, bootstrapping trust is caused by insufficient information about the newcomer services.
4. Deriving trust for the composite services was often algorithmically complex and lacked global generalization capability.

This work incorporates the solutions to multiple major challenges, and our goal is to develop a comprehensive and unified Web service trust evaluation approach. The proposed approach has the following advantages:

1. The proposed approach covers three phases of trust development, including the initial trust establishment phase, the trust improvement phase, and the trust stabilization phase, and involves two main architectures of Web services, namely, single architecture and composition architecture.
2. The proposed approach fully considers the subjective initiative of customers, involves not only their preferences but also their expectations, and leaves the setting of algorithm parameters to customers, realizing the personalization of trust evaluation.
3. Bootstrapping trust in the proposed approach cleverly utilizes customer expectations without any priori information and only needs to set the distribution of virtual ratings according to the maximum entropy principle.
4. In this work, the principle of deriving trust for composite services is simple to understand, the characteristics are stable, the algorithm is easy to implement, and the method has good global generalization ability.

## 4. Trust Evaluation Model

### 4.1. Trust Specification

Researchers have begun to recognize that Web service trust is an important attribute to measure and evaluate. Based on exploration and analysis of trust literature and extension of the online trust principles, Aljazzaf et al. presented some trust principles that form the basis of trust in Web services [54]. After a comprehensive reference to other literature [55,56], we believe that a Web service trust model should consider at least the following trust principles:

- Trust is based on information: There is a need to know information about a Web service to establish trust.
- Dynamic nature of trust: Trust is dynamic and changes over time and with future experiences. This requires a continuous evaluation of Web services’ trustworthiness.
- Trust and identity: Trust depends on identity. Having identity enables the past experience of the interactions to be built on and mapped to that identity.
- Trust is based on context: Trust is not a fixed value connected with a Web service; rather, it is dependent on the performance of the service and only applies in a certain context at a specific time.
- Categories of trust semantics: Semantic characteristics of trust ratings are important to interpret the meaning of those measurements.
- Individual and collective ratings: Considering individual and collective ratings to predict the trust in Web services helps the customer to make a better selection decisions.
- Customer expectations: Trust is not absolute but a matter of degree. Therefore, a trust-based system helps the customer to select a Web service based on the customer’s expectations.
- Trust development phases: Trust in Web services should consider the three trust development phase (initial trust establishment, trust improvement, and trust stabilization).
- Trust approaches: Using a hybrid of multiple trust methods results in a better and more robust method to model trust in Web services.

Our previous work has addressed some of the above Web service trust principles. This work is a further extension of the previous work and will address all the above principles. To facilitate description, we firstly defines some symbols, as shown in Symbol description list 1.

**Symbol description list 1**:

1. $s_{i}\left(i=1,\ldots ,I\right)$ represents a concrete element in a service market.
2. $c_{l}\left(l=1,\ldots ,L\right)$ represents a concrete customer in a community.
3. Direct trust is personal and subjective, representing an individual’s rating on a Web service. $t_{l\to i}^{s}$, which is in the range of $\left[0,1\right]$, represents customer $c_{l}$’s direct trust in service $s_{i}$.
4. Indirect trust is the collective view of a Web service’s character or standing. The collective view synthesizes the ratings submitted by the customers who have been interacted with the service. $T_{i}$, which is called indirect trust in $s_{i}$, represents the aggregation of individual direct trust in $s_{i}$ and is processed as a random variable in the range of $\left[0,1\right]$.

$t_{l\to i}^{s}$ is given subjectively by $c_{l}$ according to his/her historical interactions with $s_{i}$, which evolves over time and with new interactions. If $c_{l}$ has engaged with $s_{i}$ and has given it a rating, $t_{l\to i}\neq null$; otherwise, $t_{l\to i}=null$. $c_{l}$ must keep updating his/her rating on $s_{i}$ in response to the successive QoS of $s_{i}$ supplied to him/her. The subjective evaluation disparities in the ratings on $s_{i}$ submitted by different customers represent the cognitive differences and preferences of these customers. $T_{i}$ is relatively objective and represents a collective evaluation of a group of customers. The ratings on $s_{i}$ submitted by customers are seen as samples of the random variable $T_{i}$. In theory, $T_{i}$ should be continuous.

Due to the differences in characteristics between direct and indirect trust, this work expresses direct and indirect trust in distinct forms. This work tries to combine these two trust expressive forms to create a stronger and more comprehensive way for evaluating trust in Web services.

The process of selecting a service has evolved into a comparison of Web service trust. Because direct trust values are expressed as determinate values with a fairly basic comparison procedure, this work does not go into detail. It is worth noting that indirect trust values are random variables that can’t be directly compared as determinate values. The statistical features of indirect trust values should be compared by service customers when comparing candidate services.

$s_{a}$ and $s_{a^{\prime }}$ are two Web services that perform the same task with the indirect trust values $T_{a}$ and $T_{a^{\prime }}$. Both $T_{a}$ and $T_{a^{\prime }}$ are continuous random variables with values in the $\left[0,1\right]$ range. To compare random variables $T_{a}$ and $T_{a^{\prime }}$, a variety of methods can be employed, the most popular of which is to compare the expected values of $T_{a}$ and $T_{a^{\prime }}$, that is, $T_{a}>T_{a^{\prime }}$ if and only if $E\left[T_{a}\right]>E\left[T_{a^{\prime }}\right]$. In this work, a novel random variable comparison method is employed, as indicated in Symbol description list 2.

**Symbol description list 2**:

5. Expected trust level α is the lowest degree of trust that a consumer can expect from a Web service’s competency in real-world situations.
6. If $Pr\left(T_{a}⩾\alpha \right)>Pr\left(T_{a^{\prime }}⩾\alpha \right)$, it is said that $T_{a}$ is greater than $T_{a^{\prime }}$ and $s_{a}$ is more trustworthy than $s_{a^{\prime }}$ for an expected trust level $\alpha \in \left[0,1\right]$. These two relationships are symbolically represented by the formulas $\nabla_{\alpha }\left(T_{a}\right)>\nabla_{\alpha }\left(T_{a^{\prime }}\right)$ and $\nabla_{\alpha }\left(s_{a}\right)≻\nabla_{\alpha }\left(s_{a^{\prime }}\right)$, respectively.
7. The probability density functions of $T_{a}$ and $T_{a^{\prime }}$ are $f_{a}\left(x\right)$ and $f_{a^{\prime }}\left(x\right)$, respectively. $\nabla_{\alpha }\left(T_{a}\right)>\nabla_{\alpha }\left(T_{a^{\prime }}\right)$ can be converted to $\int_{\alpha }^{1}f_{a}\left(x\right)\;dx>\int_{\alpha }^{1}f_{a^{\prime }}\left(x\right)\;dx$.

In different contexts, there may be different trust evaluation strategies [57], and different customers have different trust expectations for Web services, so the expected trust level α should be determined by the specific context and customer requirements. By setting different expected trust levels, the service comparison method enables personalized service selection because, for different expected trust levels, the results of the service comparison may be different.

This method of comparison seems very abstract, but in fact, we use it all the time. Many e-commerce sites such as Amazon now have customer ratings of the items they buy. These ratings are generally set at five levels, one to five stars. Customers who want to buy the items can refer to these ratings. Some customers pay attention to the proportion of four-star and above ratings, while others pay more attention to the proportion of five-star ratings. This reflects that different customers have different expectations of the items.

### 4.2. Deriving Trust for Composite Services

A composite service’ trustworthiness is determined by the trustworthiness of its underlying component services [58]. A service composition system that may use the trustworthiness of separate components to evaluate the composite service’s derived trustworthiness is currently a work in progress. This is due in part to the lack of a flexible trust evaluation paradigm. This challenge is addressed by the trust evaluation approach given in this work.

“A chain is no stronger than its weakest link”, says an English proverb. Any system has a common characteristic in that different parts contribute differently to the system’s performance, but the weakest part often determines the overall system’s reliability. The whole is only as reliable as its weakest part. Similarly, a composite service is only as trustworthy as its least trustworthy component service.

**Figure 2:**
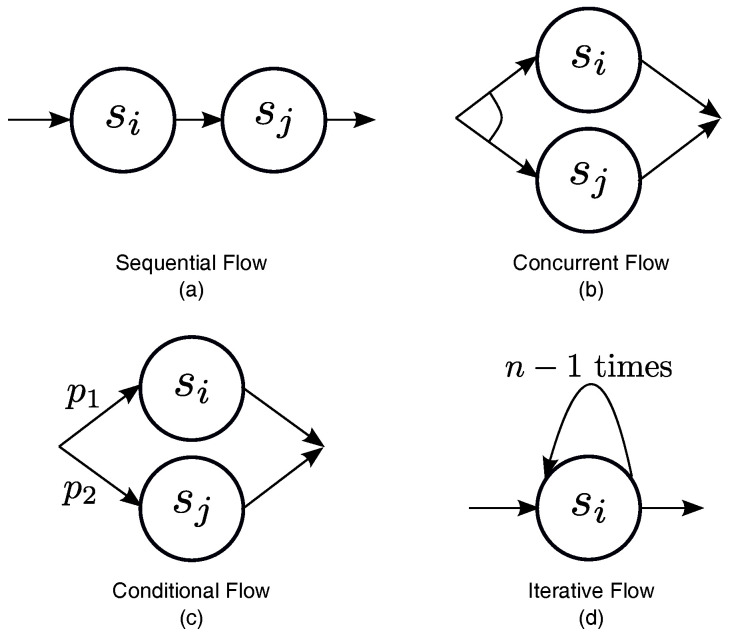
Four typical control flows appearing in composition.

In order to evaluate the trustworthiness of composite services, this work investigates four types of typical control flows that are frequently encountered in service compositions (see Figure 2) [9,19,50,59,60,61]. For the sake of simplicity, this work considers a composition of two Web services, $s_{i}$ and $s_{j}$, with the indirect trust values, $T_{i}$ and $T_{j}$, respectively. $T_{i}$ and $T_{j}$ are continuous random variables with probability density functions $f_{i}\left(x\right)$ and $f_{j}\left(x\right)$, respectively, in the range $\left[0,1\right]$. $\int_{0}^{1}f_{i}\left(x\right)\;dx=1$ and $\int_{0}^{1}f_{j}\left(x\right)\;dx=1$ are simple to find. $s_{C}$ stands for the composite service, and $T_{C}$ stands for its indirect trust value. It is reasonable to assume that $T_{C}$ is a continuous random variable with the probability density function $f_{C}\left(x\right)$ as well.

(a) Sequential Flow: Define ⊙ as a sequence operator. $s_{C}=s_{i}⊙s_{j}$ represents that the composite service $s_{C}$ executes the component service $s_{i}$ before moving on to the component service $s_{j}$. In a sequential flow (see Figure 2a), each component service must be executed. According to the previously described idea about how to evaluate the trustworthiness of composite services, $f_{C}\left(x\right)$ can be computed as follows:
  (1) $$\begin{array}{c} \begin{array}{cc} f_{C}\left(x\right) & =f_{i}\left(x\right)⊙f_{j}\left(x\right)\\ \; & =\int_{x}^{1}f_{i}\left(x\right)f_{j}\left(u\right)\;du+\int_{x}^{1}f_{i}\left(v\right)f_{j}\left(x\right)\;dv. \end{array} \end{array}$$
  The above formula shows that the composite service is only as trustworthy as its least trustworthy component service.
(b) Concurrent Flow: Define ‖ as a parallel operator. $s_{C}=s_{i}\|s_{j}$ represents that the composite service $s_{C}$ executes both component service $s_{i}$ and component service $s_{j}$ concurrently. In a concurrent flow (see Figure 2b), like a sequential flow, each of the component services must be executed, existing only in the difference of execution sequence. As a result, $f_{C}\left(x\right)$ is computed in the same way as $f_{C}\left(x\right)$ for a sequential flow, i.e.:
  (2) $$\begin{array}{c} \begin{array}{cc} f_{C}\left(x\right) & =f_{i}\left(x\right)\|f_{j}\left(x\right)=f_{i}\left(x\right)⊙f_{j}\left(x\right)\\ \; & =\int_{x}^{1}f_{i}\left(x\right)f_{j}\left(u\right)\;du+\int_{x}^{1}f_{i}\left(v\right)f_{j}\left(x\right)\;dv. \end{array} \end{array}$$
(c) Conditional Flow: Define ⊕ as a choice operator. $s_{C}=s_{i}⊕s_{j}$ represents that the composite service $s_{C}$ behaves as either component service $s_{i}$ or service $s_{j}$. As shown in Figure 2c, any one of the component services is executed in a conditional flow. For example, let $s_{i}$ and $s_{j}$ be followed with executed probabilities, $p_{1}$ and $p_{2}$$\left(p_{1}+p_{2}=1\right)$, respectively. By examining the composite task, these probability can be anticipated. $f_{C}\left(x\right)$ can be thought of as a weighted sum of $f_{i}\left(x\right)$ and $f_{j}\left(x\right)$, i.e.:
  (3) $$\begin{array}{cc} f_{C}\left(x\right) & =f_{i}\left(x\right)⊕f_{j}\left(x\right)=p_{1}\times f_{i}\left(x\right)+p_{2}\times f_{j}\left(x\right). \end{array}$$
(d) Iterative Flow: Define σ as an iteration operator. $s_{C}=\sigma_{n}\left(s_{i}\right)$ represents that the composite service $s_{C}$ executes *n* times the component service $s_{i}$. In an iterative flow, the component service must be executed similarly to a sequential flow. The difference is that the service is executed *n* times instead of just once (see Figure 2d). The value of *n* may not be constant and may vary depending on the runtime situation. The procedure for computing $f_{C}\left(x\right)$ is similar to that for a sequential flow, except ⊙ must be applied n−1 times iteratively, as follows:
  (4) $$\begin{array}{cc} f_{C}\left(x\right) & =\sigma_{n}\left(f_{i}\left(x\right)\right)=\underset{n-1\;times\;⊙}{\underset{︸}{f_{i}\left(x\right)⊙f_{i}\left(x\right)⊙\cdots ⊙f_{i}\left(x\right)}}. \end{array}$$

**Theorem** **1.** *Consider a composition of two Web services, $s_{i}$ and $s_{j}$, with indirect trust values, $T_{i}$ and $T_{j}$, respectively. For an expected trust level, $\alpha \in \left[0,1\right]$, there are:*

$$\nabla_{\alpha }\left(T_{i}⊙T_{j}\right)=\nabla_{\alpha }\left(T_{i}\right)\times \nabla_{\alpha }\left(T_{j}\right),\;\nabla_{\alpha }\left(T_{i}\|T_{j}\right)=\nabla_{\alpha }\left(T_{i}\right)\times \nabla_{\alpha }\left(T_{j}\right),\;\nabla_{\alpha }\left(T_{i}⊕T_{j}\right)=p_{1}\times \nabla_{\alpha }\left(T_{i}\right)+p_{2}\times \nabla_{\alpha }\left(T_{j}\right),\;\mathrm{and}\;\nabla_{\alpha }\left(\sigma_{n}\left(T_{i}\right)\right)=\underset{n-1\;times\;\times }{\underset{︸}{\nabla_{\alpha }\left(T_{i}\right)\times \nabla_{\alpha }\left(T_{i}\right)\times \cdots \times \nabla_{\alpha }\left(T_{i}\right)}}.$$

**Proof of Theorem** **1.** In a sequential flow or concurrent flow, $f_{C}\left(x\right)=\int_{x}^{1}f_{i}\left(x\right)f_{j}\left(u\right)\;du+\int_{x}^{1}f_{i}\left(v\right)f_{j}\left(x\right)\;dv$, thus:

$$\begin{array}{c} \begin{array}{cc} \int_{\alpha }^{1}f_{C}\left(x\right)\;dx= & \int_{\alpha }^{1}\left(\int_{x}^{1}f_{i}\left(x\right)f_{j}\left(u\right)\;du+\int_{x}^{1}f_{i}\left(v\right)f_{j}\left(x\right)\;dv\right)\;dx\\ = & \int_{\alpha }^{1}(f_{i}\left(x\right)+f_{j}\left(x\right)-f_{i}\left(x\right)F_{j}\left(x\right)\\ \; & \;-f_{j}\left(x\right)F_{i}\left(x\right))\;dx\\ = & \int_{\alpha }^{1}f_{i}\left(x\right)\;dx+\int_{\alpha }^{1}f_{j}\left(x\right)\;dx-{\left[F_{i}\left(x\right)F_{j}\left(x\right)\right]}_{\alpha }^{1}\\ = & \int_{\alpha }^{1}f_{i}\left(x\right)\;dx+\int_{\alpha }^{1}f_{j}\left(x\right)\;dx-1\\ \; & \;+\left(1-\int_{\alpha }^{1}f_{i}\left(x\right)\;dx\right)\left(1-\int_{\alpha }^{1}f_{j}\left(x\right)\;dx\right)\\ = & \int_{\alpha }^{1}f_{i}\left(x\right)\;dx\int_{\alpha }^{1}f_{j}\left(x\right)\;dx, \end{array} \end{array}$$

i.e., $\nabla_{\alpha }\left(T_{i}⊙T_{j}\right)=\nabla_{\alpha }\left(T_{i}\right)\times \nabla_{\alpha }\left(T_{j}\right)$ and $\nabla_{\alpha }\left(T_{i}\|T_{j}\right)=\nabla_{\alpha }\left(T_{i}\right)\times \nabla_{\alpha }\left(T_{j}\right)$.

In a conditional flow, $f_{C}\left(x\right)=p_{1}\times f_{i}\left(x\right)+p_{2}\times f_{j}\left(x\right)$, thus:

$$\begin{array}{c} \begin{array}{cc} \int_{\alpha }^{1}f_{C}\left(x\right)\;dx= & \int_{\alpha }^{1}\left(p_{1}\times f_{i}\left(x\right)+p_{2}\times f_{j}\left(x\right)\right)\;dx\\ = & p_{1}\times \int_{\alpha }^{1}f_{i}\left(x\right)\;dx+p_{2}\times \int_{\alpha }^{1}f_{j}\left(x\right)\;dx, \end{array} \end{array}$$

i.e., $\nabla_{\alpha }\left(T_{i}⊕T_{j}\right)=p_{1}\times \nabla_{\alpha }\left(T_{i}\right)+p_{2}\times \nabla_{\alpha }\left(T_{j}\right)$.

In an iterative flow, it is easy to see that:

$$\begin{array}{c} \begin{array}{c} \int_{\alpha }^{1}f_{C}\left(x\right)\;dx=\underset{n}{\underset{︸}{\int_{\alpha }^{1}f_{i}\left(x\right)\;dx\cdots \int_{\alpha }^{1}f_{i}\left(x\right)\;dx}}, \end{array} \end{array}$$

i.e., $\nabla_{\alpha }\left(\sigma_{n}\left(T_{i}\right)\right)=\underset{n-1\;times\;\times }{\underset{︸}{\nabla_{\alpha }\left(T_{i}\right)\times \nabla_{\alpha }\left(T_{i}\right)\times \cdots \times \nabla_{\alpha }\left(T_{i}\right)}}$. □

In the above formulae, $F_{i}\left(x\right)$ and $F_{j}\left(x\right)$ are the cumulative distribution functions of random variables $T_{i}$ and $T_{j}$, respectively, namely, $F_{i}\left(x\right)=\int_{-\infty }^{x}f_{i}\left(v\right)\;dv=\int_{0}^{x}f_{i}\left(v\right)\;dv$ and $F_{j}\left(x\right)=\int_{-\infty }^{x}f_{j}\left(u\right)\;du=\int_{0}^{x}f_{j}\left(u\right)\;du$. No matter what kind of control flows is deployed, we can always find $\int_{0}^{1}f_{C}\left(x\right)\;dx=1$, i.e., the random variable $T_{C}$ is also in the range of $\left[0,1\right]$ like $T_{i}$ and $T_{j}$. This ensures the Web service trust evaluation model maintains consistency for both elementary services and composite services.

This paper has mathematically shown the methods to evaluate the trust of each flow. These evaluation methods can all be generalized to more than two component services in a straightforward way. In practice, a composition may consist of one or more of these basic control flows. No matter how complicated the composition is, the trustworthiness of the composition can be always evaluated.

### 4.3. Extension of Trust Evaluation Model

Customers should enter numerical values in the range of $\left[0,1\right]$ when rating $s_{i}$. In practice, however, it is more appropriate for a consumer to express his/her direct trust in $s_{i}$ through a subjective qualitative evaluation rather than a quantitative evaluation. According to previous interactions with $s_{i}$, customers make subjective qualitative ratings on $s_{i}$ initially, then convert these subjective qualitative ratings to quantitative forms. A comparison between a subjective qualitative rating and a reference value is used in the conversion. This work creates a conversion comparison table (see Table 2). Based on the semantic characteristics of these ratings, this work divides them into 11 categories, which correspond to 11 values. For example, the subjective qualitative rating “very untrustworthy” corresponds to the value 0.2, indicating that $t_{l\to i}^{s}$ is 0.2, and the subjective qualitative rating “rather trustworthy” corresponds to the value 0.7, indicating that $t_{l\to i}^{s}$ is 0.7. Note that a value in the middle, such as 0.78, indicates that $s_{i}$ has a subjective qualitative rating that is somewhere between “rather trustworthy” and “very trustworthy” but is closer to the latter.

In doing so, $T_{i}$ will be treated as a discrete random variable with 101 possible values 0,0.01,…,1.00. To make the statement easier to understand, let $x_{k}$ symbolize the potential values of $T_{i}$, namely, $x_{k}=\frac{k}{100}$ for k=0,1,2,…,100. $T_{i}$’s probability function is defined as follows:

(5) $$\begin{array}{c} \begin{array}{cc} f_{i}\left(x_{k}\right) & =Pr(T_{i}=x_{k})\\ \; & =\frac{Cnt(t_{l\to i}^{s}|t_{l\to i}^{s}=x_{k})}{Cnt(t_{l\to i}^{s}|t_{l\to i}^{s}\neq null)}, \end{array} \end{array}$$

where $x_{k}\in \left\{0,0.01,0.02,\ldots ,1\right\}$, $l\in \left\{1,2,\ldots ,L\right\}$ and $i\in \left\{1,2,\ldots ,I\right\}$.

$Cnt(t_{l\to i}^{s}|t_{l\to i}=x_{k})$ is the number of $t_{l\to i}^{s}$ when $t_{l\to i}^{s}=x_{k}$ in Formula (5). Similarly, $Cnt(t_{l\to i}^{s}|t_{l\to i}^{s}\neq null)$ is the number of $t_{l\to i}^{s}$ when $t_{l\to i}^{s}\neq null$, also known as the number of actual ratings on $s_{i}$. Because $Cnt(t_{l\to i}^{s}|t_{l\to i}^{s}\neq null)$ cannot be 0, Formula (5) is applicable to the case where ratings are required on $s_{i}$. In the discrete case, $\nabla_{\alpha }\left(T_{a}\right)>\nabla_{\alpha }\left(T_{a^{\prime }}\right)$ is equivalent to $\sum_{n=100\alpha }^{100}f_{a}\left(x_{k}\right)>\sum_{n=100\alpha }^{100}f_{a^{\prime }}\left(x_{k}\right)$, where $\alpha \in \left\{0,0.01,\ldots ,1\right\}$.

The above trust evaluation model has not taken into account the time decay of the ratings and the credibility of the raters. Older ratings are less important than newer ones since older ratings may become obsolete or meaningless as time passes. On the other hand, ratings submitted by more reliable raters are more significant. In Symbol description list 3, we have introduced some new symbols for the simplicity of expression.

**Symbol description list 3**:

8. $\eta_{i}$ represents the number of actual ratings on $s_{i}$, i.e., $\eta_{i}$=$Cnt(t_{l\to i}^{s}|t_{l\to i}^{s}\neq null)$.
9. τ represents current moment, $\tau_{l,i}$ represents the moment when $c_{l}$ last updated the rating on $s_{i}$, and $\phi_{l,i}$ represents the time interval between τ and $\tau_{l,i}$, i.e., $\phi_{l,i}=\tau -\tau_{l,i}$.
10. $H(\phi_{l,i})$, which is in the range of $\left[0,1\right]$, represents a function of $\phi_{l,i}$. The smaller $\phi_{l,i}$ is, the nearer the value of $H(\phi_{l,i})$ to unity; the larger $\phi_{l,i}$ is, the nearer the value of $H(\phi_{l,i})$ to 0. In other words, $H(\phi_{l,i})$ indicates the newness degree of $t_{l\to i}^{s}$.
11. $t_{l\to m}^{c}$, which is in the range of $\left[0,1\right]$, represents customer $c_{l}$’s trust in another customer $c_{m}$ and also refers to the preference similarity between $c_{l}$ and $c_{m}$ in $c_{l}$’s individual opinion.
12. $T_{l\to i}$ represents personalized indirect trust in $s_{i}$ from $c_{l}$’s perspective and is also processed as a random variable in the range of $\left[0,1\right]$.

$t_{l\to m}^{c}$ and $H(\phi_{l,i})$ have completely different connotations, but they both emphasize the importance of a rating. A mechanism is needed to integrate them. Referring to the literature [62], the authors define a new function *Z*, which allows customers to express different preferences for the credibility of raters and the time decay of ratings. *Z* is defined as follows:

(6) $$\begin{array}{c} \begin{array}{cc} Z_{\xi }\left(l,m,i\right) & =\frac{\left(1+\xi^{2}\right)\times t_{l\to m}^{c}\times H\left(\phi_{m,i}\right)}{\left(\xi^{2}\times t_{l\to m}^{c}\right)+H\left(\phi_{m,i}\right)}, \end{array} \end{array}$$

where ξ>0, $l,m\in \left\{1,2,\ldots ,L\right\}$ and $i\in \left\{1,2,\ldots ,I\right\}$.

ξ gauges the relative relevance of $H(\phi_{m,i})$ to $t_{l\to m}^{c}$ in Formula (6). When ξ=1, $c_{l}$ gives equal weight to $H(\phi_{m,i})$ and $t_{l\to m}^{c}$; when ξ>1, $c_{l}$ gives more weight to $H(\phi_{m,i})$; and when ξ<1, $c_{l}$ gives more weight to $t_{l\to m}^{c}$.

The probability function of $T_{l\to i}$ is defined as follows:

(7) $$\begin{array}{c} \begin{array}{cc} f_{l\to i}\left(x_{k}\right) & =Pr(T_{l\to i}=x_{k})\\ \; & =\frac{Sum(Z_{\xi }\left(l,m,i\right)|t_{m\to i}^{s}=x_{k})}{Sum(Z_{\xi }\left(l,m,i\right)|t_{m\to i}^{s}\neq null)}, \end{array} \end{array}$$

where $x_{k}\in \left\{0,0.01,0.02,\ldots ,1\right\}$, $l,m\in \left\{1,2,\ldots ,L\right\}$ and $i\in \left\{1,2,\ldots ,I\right\}$.

$Sum(Z_{\xi }\left(l,m,i\right)|t_{m\to i}^{s}=x_{k})$ is the sum of $Z_{\xi }\left(l,m,i\right)$ when $t_{m\to i}^{s}=x_{k}$ in Formula (7). Similarly, $Sum(Z_{\xi }\left(l,m,i\right)|t_{m\to i}^{s}\neq null)$ is the sum of $Z_{\xi }\left(l,m,i\right)$ when $t_{m\to i}^{s}\neq null$, also known as the number of $c_{l}$’s calculated ratings on $s_{i}$. Formula (7), like Formula (5), is applicable to the case that there must be ratings on $s_{i}$.

$s_{a}$ and $s_{a^{\prime }}$ are two Web services that perform the same task. From the perspective of $c_{l}$, $T_{l\to a}$ and $T_{l\to a^{\prime }}$ are personalized indirect trust values of $s_{a}$ and $s_{a^{\prime }}$, respectively. It is said that $T_{l\to a}$ is greater than $T_{l\to a^{\prime }}$ and $s_{a}$ is more trustworthy than $s_{a^{\prime }}$ in $c_{l}$’s viewpoint if $Pr\left(T_{l\to a}⩾\alpha \right)>Pr\left(T_{l\to a^{\prime }}⩾\alpha \right)$ for an expected trust level $\alpha \in \left\{0,0.01,0.02,\ldots ,1\right\}$. These two relationships are symbolically described by the equations $\nabla_{\alpha }\left(T_{l\to a}\right)>\nabla_{\alpha }\left(T_{l\to a^{\prime }}\right)$ and $\nabla_{\alpha }{\left(s_{a}\right)}_{l}≻\nabla_{\alpha }{\left(s_{a^{\prime }}\right)}_{l}$, respectively.

Our previous work designed a trust management mechanism based on peer-to-peer network, where each customer in the community is both a provider and a requester of ratings. This mechanism makes it easy to store and manage $t_{l\to m}^{c}$, $t_{m\to i}^{s}$ and $\tau_{m,i}$, and allows flexible and robust support for personalized trust evaluation.

### 4.4. Bootstrapping Trust for Newcomer Services

Trust in Web services is established based on the historical ratings. It is tough to give a recommendation for a new service if no ratings are available [13] because there is usually no way to judge its initial trust value [42,44,45]. As a result, the new service cannot be compared with other services. The new service should be given an initial trust value. Existing services will be disadvantaged if the initial trust value is high, whereas a low initial trust value will deter new services from competing [42]. Furthermore, if a system relies on a few ratings to establish trust, the subjectivities of the raters may have an undue influence. One of the first issues service selection must address is how to allow a new service to compete. Introducing virtual ratings can effectively solve this problem. Virtual ratings are not real user ratings, and their introduction aims at constructing the indirect trust probability distribution for a new service. The values of the virtual ratings are hypothetical, i.e., possible values of user ratings in the absence of the a priori information available.

According to the law of large numbers, the frequency of a random event, as the number of trials increases, tends to a stable value. That is, plenty of ratings on $s_{i}$ can effectively and objectively reflect the statistical properties of $T_{l\to i}$. However, the question is, how much is plenty? There is no one-size-fits-all response to this question. The statistical features of $T_{l\to i}$ can be portrayed more effectively and objectively the more calculated ratings on $s_{i}$. Plenty is a fuzzy term similar to trust in that it is not a precise amount. In order to simplify the expression, we define some new symbols in Symbol description list 4.

**Symbol description list 4**:

13. Γ represents the set containing all non-negative numbers, while $\gamma_{l\to i}$ represents the number of $c_{l}$’s calculated ratings on $s_{i}$, i.e., $\gamma_{l\to i}$=$Sum(Z_{\xi }\left(l,m,i\right)|t_{m\to i}^{s}\neq null)$ and $\gamma_{l\to i}\in \Gamma$.
14. Plenty, denoted by $\tilde{P}$, is called a fuzzy set defined on Γ, which has a membership function $\mu_{\tilde{P}}:\Gamma \to \left[0,1\right]$. For each $\gamma_{l\to i}\in \Gamma$, $\gamma_{l\to i}$ is called a fuzzy member of $\tilde{P}$. The value $\mu_{\tilde{P}}\left(\gamma_{l\to i}\right)$ is called the grade of membership of $\gamma_{l\to i}$ in $\tilde{P}$.

It is self-evident that the larger $\gamma_{l\to i}$, the more $\gamma_{l\to i}$ belongs to $\tilde{P}$. The grade of membership of 0 in $\tilde{P}$ is 0, and the grade of membership of +∞ in $\tilde{P}$ is 1. There is no one-size-fits-all way to create fuzzy membership functions. It is possible that more than one function can be utilized as the fuzzy membership function of $\tilde{P}$. This work simply chooses one of these based on the present application needs. The function that has been chosen is as follows:

(8) $$\begin{array}{c} \mu_{\tilde{P}}\left(\gamma_{l\to i}\right)=1-\beta^{\gamma_{l\to i}}\;\left(0<\beta <1\right). \end{array}$$

**Figure 3:**
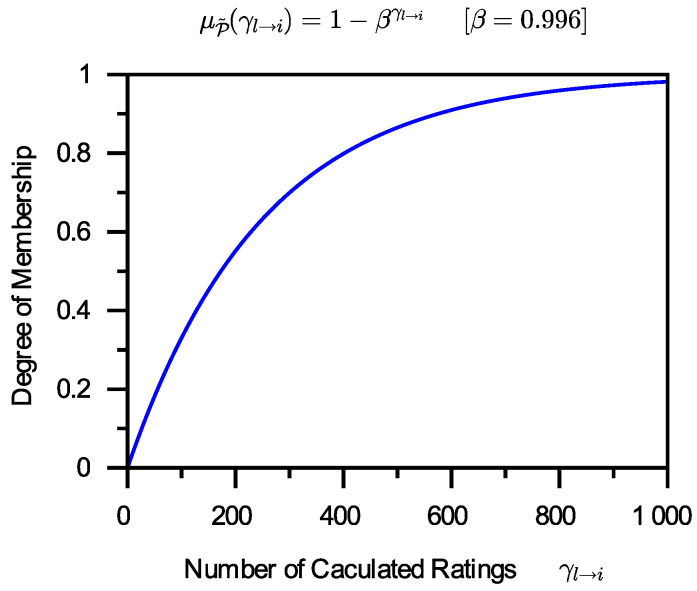
A membership function of $\tilde{P}$.

In Formula (8), β is a parameter of the membership function. If $c_{l}$ presets β to various values, the function curve will be varied. Smaller β values can result in faster changes, whereas bigger β values can result in slower changes. However, the curve change trend is essentially the same. One instance of this function is shown in Figure 3.

The goal of introducing virtual ratings is to build the probability distribution of $T_{l\to i}$ for the new service $s_{i}$ that has few or no calculated ratings. If there are too many virtual ratings on $s_{i}$ introduced, the calculated ratings on $s_{i}$ will be buried; if there are too few virtual ratings on $s_{i}$ introduced, virtual ratings will not be able to play the role they should. The more calculated ratings on $s_{i}$, the fewer virtual ratings on $s_{i}$ should be introduced. Of course, fewer is in relation to the number of calculated ratings on $s_{i}$. Assume that there are $\gamma_{l\to i}$ calculated ratings on $s_{i}$ and that $\delta_{l\to i}$ virtual ratings on $s_{i}$ should be introduced. It is reasonable to suppose that $\delta_{l\to i}$ is associated with $\gamma_{l\to i}$ and $\mu_{\tilde{P}}\left(\gamma_{l\to i}\right)$. In the case where there are $\gamma_{l\to i}$ calculated ratings on $s_{i}$, this work recommends that $\left(\gamma_{l\to i}+101\right)\beta^{\gamma_{l\to i}}$ virtual ratings on $s_{i}$ should be introduced, i.e.:

(9) $$\begin{array}{c} \begin{array}{cc} \delta_{l\to i} & =\left(\gamma_{l\to i}+101\right)\left(1-\mu_{\tilde{P}}\left(\gamma_{l\to i}\right)\right)\\ \; & =\left(\gamma_{l\to i}+101\right)\beta^{\gamma_{l\to i}}. \end{array} \end{array}$$

**Figure 4:**
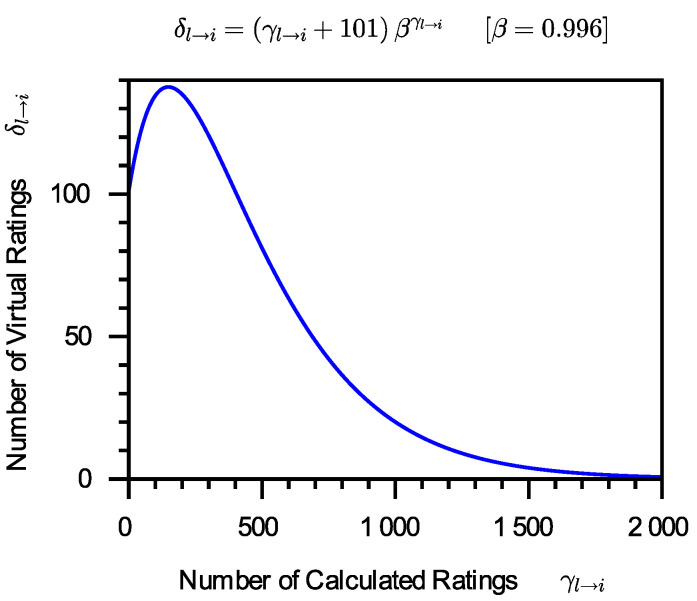
The number of virtual ratings which should be introduced.

Figure 4 depicts the relationship between the number of calculated ratings on $s_{i}$ and the number of virtual ratings on $s_{i}$. When the number of calculated ratings on $s_{i}$ increases from 0, the number of virtual ratings on $s_{i}$ that need be introduced also increases from 101. The number of virtual ratings on $s_{i}$ reaches a peak as the number of calculated ratings on $s_{i}$ grows, then progressively drops until it is near to 0. When the number of calculated ratings on $s_{i}$ is minimal, constructing probability function of $T_{l\to i}$ needs to introduce virtual ratings on $s_{i}$; when the number of calculated ratings on $s_{i}$ is large, these calculated ratings on $s_{i}$ can construct probability function of $T_{l\to i}$ effectively without the use of virtual ratings on $s_{i}$. Figure 4 shows a curve changing trend that meets our perception of this issue.

The next issue is how to set values for virtual ratings on $s_{i}$. There is no reason to prefer one possible value over the others. The only plausible assumption is that each possible virtual rating value is assigned the same probability. This assumption is consistent with the maximum entropy principle. The essence of maximum entropy principle is that the most reasonable inference about the unknown distribution, given the known partial knowledge, is the most uncertain or random inference, which is the unbiased choice we can make. Under this assumption, $\delta_{l\to i}$ should be evenly divided into 101 smaller numbers. The probability function of $T_{l\to i}$ in Formula (7) can be redefined as follows:

(10) $$\begin{array}{c} \begin{array}{cc} f_{l\to i}\left(x_{k}\right) & =Pr(T_{l\to i}=x_{k})\\ \; & =\frac{Sum\left(Z_{\xi }\left(l,m,i\right)|t_{m\to i}^{s}=x_{k}\right)+\frac{\delta_{l\to i}}{101}}{Sum\left(Z_{\xi }\left(l,m,i\right)|t_{m\to i}^{s}\neq null\right)+\delta_{l\to i}}. \end{array} \end{array}$$

### 4.5. Two Corollaries

A question to examine in service composition is how to ensure that the composite service composed of optimal component services is also optimal. Formula (1) can be rewritten as follows in the discrete case:

(11) $$\begin{array}{c} \begin{array}{cc} f_{l\to C}\left(x_{k}\right) & =f_{l\to i}\left(x_{k}\right)⊙f_{l\to j}\left(x_{k}\right)\\ & =\sum_{u=k}^{100}f_{l\to i}\left(x_{k}\right)f_{l\to j}\left(x_{u}\right)+\sum_{v=k+1}^{100}f_{l\to i}\left(x_{v}\right)f_{l\to j}\left(x_{k}\right). \end{array} \end{array}$$

According to Theorem 1 and Formula (11), the following equation can be found:

(12) $$\begin{array}{c} \begin{array}{c} \sum_{k=100\alpha }^{100}f_{l\to C}\left(x_{k}\right)=\sum_{k=100\alpha }^{100}\left(f_{l\to i}\left(x_{k}\right)⊙f_{l\to j}\left(x_{k}\right)\right)=\sum_{k=100\alpha }^{100}f_{l\to i}\left(x_{k}\right)\sum_{k=100\alpha }^{100}f_{l\to j}\left(x_{k}\right). \end{array} \end{array}$$

Because $\sum_{k=100\alpha }^{100}f_{l\to i}\left(x_{k}\right)⩽1$ and $\sum_{k=100\alpha }^{100}f_{l\to j}\left(x_{k}\right)⩽1$, we can find $\sum_{k=100\alpha }^{100}f_{l\to C}\left(x_{k}\right)⩽\sum_{k=100\alpha }^{100}f_{l\to i}\left(x_{k}\right)$ and $\sum_{k=100\alpha }^{100}f_{l\to C}\left(x_{k}\right)⩽\sum_{k=100\alpha }^{100}f_{l\to j}\left(x_{k}\right)$, i.e., $\nabla_{\alpha }\left(T_{l\to i}⊙T_{l\to j}\right)⩽\nabla_{\alpha }\left(T_{l\to i}\right)$ and $\nabla_{\alpha }\left(T_{l\to i}⊙T_{l\to j}\right)⩽\nabla_{\alpha }\left(T_{l\to j}\right)$.

As the number of component services in a composite service grows, the composite service’s trustworthiness may deteriorate. People’s perceptions are compatible with this finding. The following two corollaries can be found based on the prior discussion and Theorem 1:

**Corollary** **1.** *Let $s_{a}$ and $s_{a^{\prime }}$, $s_{b}$ and $s_{b^{\prime }}$ be Web services offering the same function with $c_{l}$’s indirect trust values $T_{l\to a}$ and $T_{l\to a^{\prime }}$, $T_{l\to b}$ and $T_{l\to b^{\prime }}$ respectively. For an expected trust level $\alpha \in \left\{0,0.01,\ldots ,1\right\}$, if $\nabla_{\alpha }{\left(s_{a}\right)}_{l}≻\nabla_{\alpha }{\left(s_{a^{\prime }}\right)}_{l}$ and $\nabla_{\alpha }{\left(s_{b}\right)}_{l}≻\nabla_{\alpha }{\left(s_{b^{\prime }}\right)}_{l}$, then*

$$\nabla_{\alpha }{\left(s_{a}⊙s_{b}\right)}_{l}≻\nabla_{\alpha }{\left(s_{a^{\prime }}⊙s_{b^{\prime }}\right)}_{l},\;\nabla_{\alpha }{\left(s_{a}\|s_{b}\right)}_{l}≻\nabla_{\alpha }{\left(s_{a^{\prime }}\|s_{b^{\prime }}\right)}_{l},\;\nabla_{\alpha }{\left(s_{a}⊕s_{b}\right)}_{l}≻\nabla_{\alpha }{\left(s_{a^{\prime }}⊕s_{b^{\prime }}\right)}_{l},\;\nabla_{\alpha }{\left(\sigma_{n}\left(s_{a}\right)\right)}_{l}≻\nabla_{\alpha }{\left(\sigma_{n}\left(s_{a^{\prime }}\right)\right)}_{l},\mathrm{and}\nabla_{\alpha }{\left(\sigma_{n}\left(s_{b}\right)\right)}_{l}≻\nabla_{\alpha }{\left(\sigma_{n}\left(s_{b^{\prime }}\right)\right)}_{l}.$$

**Corollary** **2.** *Consider a composition of two Web services, $s_{i}$ and $s_{j}$, with $c_{l}$’s indirect trust values $T_{l\to i}$ and $T_{l\to j}$ respectively. For an expected trust level $\alpha \in \left\{0,0.01,\ldots ,1\right\}$,*

$$\nabla_{\alpha }\left(T_{l\to i}⊙T_{l\to j}\right)⩽\nabla_{\alpha }\left(T_{l\to i}\right),\nabla_{\alpha }\left(T_{l\to i}⊙T_{l\to j}\right)⩽\nabla_{\alpha }\left(T_{l\to j}\right),\;\nabla_{\alpha }\left(T_{l\to i}\|T_{l\to j}\right)⩽\nabla_{\alpha }\left(T_{l\to i}\right),\nabla_{\alpha }\left(T_{l\to i}\|T_{l\to j}\right)⩽\nabla_{\alpha }\left(T_{l\to j}\right),\;\mathrm{and}\nabla_{\alpha }\left(\sigma_{n}\left(T_{l\to i}\right)\right)⩽\nabla_{\alpha }\left(T_{l\to i}\right).$$

## 5. Case Study

Our previous work has shown the feasibility of the proposed approach, which models the social features of trust in the sector of Web services and can be exploited to personalize service selection. This study will not repeat the feasibility of the proposed approach but will instead undertake a case study to illustrate the details of bootstrapping trust for the newcomer services and deriving trust for the composite services so that they may be graphically described. The case study is programmed with Scilab and MySQL, and executed on a MacBook Pro computer with the following confgurations: Intel Core i5-4308U CPU, 8 GB RAM, and Windows 10 operating system.

To the best of our knowledge, no dataset exists that perfectly matches the proposed approach. We used some synthetic data based on the extended Epinions dataset, which is a real-world dataset from Trustlet (http://www.trustlet.org/extended_epinions.html, accessed on 19 December 2021) [63,64]. Trustlet.org is a wiki-based platform for open trust metric research with the purpose of collecting and disseminating trust network datasets and trust metric scripts to facilitate comparing different trust metric algorithms. Epinions is a website where people can review products. Customers can sign up for free and write subjective reviews about many different types of items. The extended Epinions dataset contains 13,668,320 customer ratings of items, in addition to the time when these ratings were last modified and the trust or distrust values that one customer has for other customers. In general, the extended Epinions dataset is more suitable for the proposed approach, but some transformations and modifications are still needed.

In the extended Epinions dataset, the values of original ratings are 1–5, which we need to transform to within the interval [0, 1]. The approach we take is to first correspond and convert 1, 2, 3, 4, and 5 to 0.1, 0.3, 0.5, 0.7, and 0.9, respectively. Then, we calculate the mean and variance of the post-transformation rating values of a given item and use them to estimate the parameters of the Beta distribution. Finally, the Beta distribution is used to generate the same number of generated ratings as the original ratings. These generated ratings are the final values we need. We randomly replace the original ratings with the generated ratings. The original trust value of a customer to another customer is 1, and the original distrust value is −1. We modify the distrust value to 0 and set the non-existent trust value of a customer to another customer to 0.5.

Assume that the customer with MEMBER_ID 239694 is the current customer $c_{l}$. Consider the sequential flow consisting of $Task_{2}$ and $Task_{3}$ in Figure 1. The composite task performed by the sequential flow is denoted as $Task_{C}$. There are a set of candidate Web services $\left\{s_{3},s_{4},s_{5}\right\}$ that can be used to perform $Task_{2}$, while there is only one candidate Web service $s_{6}$ that can be used to perform $Task_{3}$. $c_{l}$ needs to select one in $S_{2}$ for deployment. $s_{3}$ is a new service so there is no actual rating on it. The only candidate service $s_{6}$ for $Task_{3}$ naturally is selected to deploy. Assume that the item with OBJECT_ID 18887577220 is $s_{4}$, the item with OBJECT_ID 17124462212 is $s_{5}$, and the item with OBJECT_ID 1549857 is $s_{6}$. They have 244, 125, and 1195 actual ratings, respectively. The latest date in the extended Epinions dataset is 2003-08-12, which we have taken as the current time τ. The unit for time interval $\phi_{l,i}$ is days, and the function $H(\phi_{l,i})$ is set to $0.998^{\phi_{l,i}}$, i.e., $H\left(\phi_{l,i}\right)=0.998^{\phi_{l,i}}$. The parameter ξ of $Z_{\xi }\left(l,m,i\right)$ is set to 0.3, indicating that $c_{l}$ considers $t_{l\to m}^{c}$ to be more important than $H(\phi_{m,i})$.

**Figure 5:**
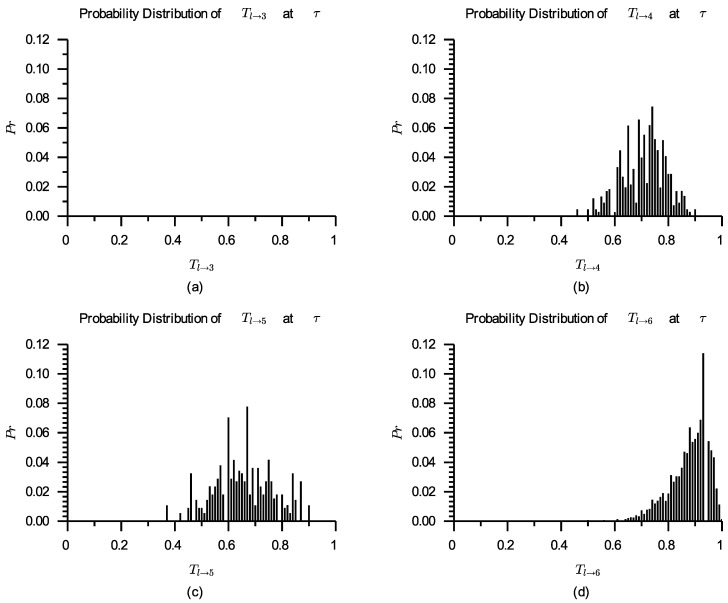
Probability distributions of $T_{l\to 3}$ (**a**), $T_{l\to 4}$ (**b**), $T_{l\to 5}$ (**c**), and $T_{l\to 6}$ (**d**) originally.

After calculating according to Formula (7), the original probability distributions for $T_{l\to 3}$, $T_{l\to 4}$, $T_{l\to 5}$, and $T_{l\to 6}$ are shown in Figure 5.

As can be seen from Figure 5, $s_{3}$ has no actual and calculated ratings and hence cannot participate in the competition; the actual and calculated rating numbers for both $s_{4}$ and $s_{5}$ are low, which can not effectively reflect their actual quality. In this instance, we must incorporate virtual ratings into the service selection process. Comparison of the number of actual ratings, the number of calculated ratings and the number of virtual ratings about the four services is shown in Table 3.

**Figure 6:**
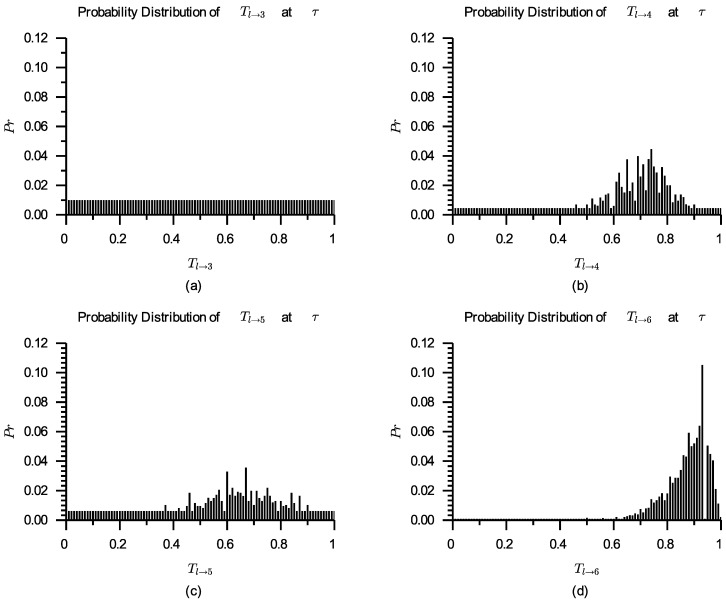
Probability distributions of $T_{l\to 3}$ (**a**), $T_{l\to 4}$ (**b**), $T_{l\to 5}$ (**c**) and $T_{l\to 6}$ (**d**) after introducing virtual ratings.

After introducing virtual ratings, the probability distributions of $T_{l\to 3}$, $T_{l\to 4}$, $T_{l\to 5}$, and $T_{l\to 6}$ are shown in Figure 6. From Figure 6, it can be seen that virtual ratings’ effect on the probability distribution of $T_{l\to i}$ reduces with the increasing number of calculated ratings on $s_{i}$. $s_{3}$ is at initial trust establishment phase, while $s_{4}$ and $s_{5}$ are at trust improvement phase. It can be considered that $s_{6}$ is coming to trust stabilization phase. The comparisons of $s_{3}$, $s_{4}$, and $s_{5}$ are shown in Table 4.

When α=0.7, there is $\nabla_{0.7}\left(T_{l\to 4}\right)>\nabla_{0.7}\left(T_{l\to 5}\right)>\nabla_{0.7}\left(T_{l\to 3}\right)$, so $c_{l}$ can find $\nabla_{0.7}{\left(s_{4}\right)}_{l}≻\nabla_{0.7}{\left(s_{5}\right)}_{l}≻\nabla_{0.7}{\left(s_{3}\right)}_{l}$; when α=0.8, there is $\nabla_{0.8}\left(T_{l\to 3}\right)>\nabla_{0.8}\left(T_{l\to 5}\right)>\nabla_{0.8}\left(T_{l\to 4}\right)$, so $c_{l}$ can find $\nabla_{0.8}{\left(s_{3}\right)}_{l}≻\nabla_{0.8}{\left(s_{5}\right)}_{l}≻\nabla_{0.8}{\left(s_{4}\right)}_{l}$.

This case study shows that for different expected trust levels, the services selected in the service selection process may be different, i.e., the optimal service is relatively optimal, rather than absolutely optimal. This case study also shows that a Web service with few or no ratings can participate in the competition and has the opportunity to be selected as the optimal service. This result accords with the fact that a consumer may try to select a new service when the existing services do not meet his/her expectations well, even though there is a decision-making risk.

**Figure 7:**
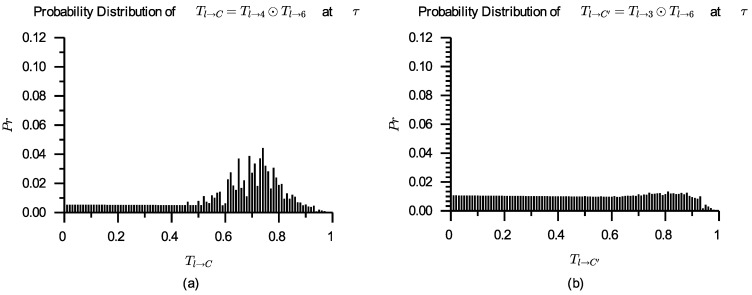
Probability distributions of $T_{l\to C}$ (**a**) and $T_{l\to C^{\prime }}$ (**b**) derived by $T_{l\to 4}⊙T_{l\to 6}$ and $T_{l\to 3}⊙T_{l\to 6}$, respectively.

As mentioned above, $s_{6}$ has been selected to perform $Task_{3}$. If $c_{l}$ set α=0.7, $s_{4}$ will be selected to perform $Task_{2}$, then there is a composite service $s_{C}=s_{4}⊙s_{6}$. In the same way, if $c_{l}$ set α=0.8, there is a composite service $s_{C^{\prime }}=s_{3}⊙s_{6}$. The probability distributions of $T_{l\to C}$ and $T_{l\to C^{\prime }}$ that are derived according to Formula (11) are shown in Figure 7, and the comparisons of $s_{C}$ and $s_{C^{\prime }}$ are shown in Table 5.

When α=0.7, there is $\nabla_{0.7}\left(T_{l\to C}\right)>\nabla_{0.7}\left(T_{l\to C^{\prime }}\right)$, so $c_{l}$ can find $\nabla_{0.7}{\left(s_{C}\right)}_{l}≻\nabla_{0.7}{\left(s_{C^{\prime }}\right)}_{l}$; when α=0.8, there is $\nabla_{0.8}\left(T_{l\to C^{\prime }}\right)>\nabla_{0.8}\left(T_{l\to C}\right)$, so $c_{l}$ can find $\nabla_{0.8}{\left(s_{C^{\prime }}\right)}_{l}≻\nabla_{0.8}{\left(s_{C}\right)}_{l}$.

From Table 4 and Table 5, the following phenomena can be seen. For a given expected trust level $\alpha \in \left[0,1\right]$ and the same service $s_{6}$, if $\nabla_{\alpha }{\left(s_{4}\right)}_{l}≻\nabla_{\alpha }{\left(s_{3}\right)}_{l}$, there is $\nabla_{\alpha }{\left(s_{4}⊙s_{6}\right)}_{l}≻\nabla_{\alpha }{\left(s_{3}⊙s_{6}\right)}_{l}$; if $\nabla_{\alpha }{\left(s_{3}\right)}_{l}≻\nabla_{\alpha }{\left(s_{4}\right)}_{l}$, there is $\nabla_{\alpha }{\left(s_{3}⊙s_{6}\right)}_{l}≻\nabla_{\alpha }{\left(s_{4}⊙s_{6}\right)}_{l}$, i.e., if the component services selected are optimal, the composite services containing these component services are also optimal.

It is worth noting that our proposed bootstrapping trust approach is applicable to the case when the overall rating values of the existing services are small. Only in this case, the new service is likely to be selected as the optimal service, i.e., it indicates that customers try the new service only if they are dissatisfied with the existing services. When the overall rating values of existing services are large, the new service is inhibited from competing. This is when additional mechanisms may be needed to enable the new service to be competitive.

## 6. Comparison with Other Approaches

The Web service trust evaluation approach proposed in this work is based on a new theory and focuses on personalized trust evaluation from the customers’ perspective. This work introduces an unprecedented concept of expected trust level to express the lowest degree of trust that a consumer can expect from a Web service’s competency. The approach proposed in this work is based on this concept. This concept is extremely subjective, and we cannot determine in advance the expected trust level for each customer. Customers set different trust expectations and may obtain different results. So, it is not meaningful for this approach to compare accuracy, efficiency, and other indicators with other approaches. Additionally, an exact benchmark trust value for a service does not exist. Various approaches for trust evaluation usually tackle different characteristics of trust. Trust evaluation is a socialization research method, and a feasible comparison scheme for trust evaluation approaches is to compare which approach is closer to the real society and better reflects the socialization characteristics of trust.

In the sector of Web services, reputation-based trust approaches primarily address concerns such as bootstrapping trust, rating credibility, trust dynamism, and trust derivation for composite services, among others. This work defines a set of criteria for the success and efficacy of reputation-based trust approaches targeting the sector of Web services, based on the reference [33]. Table 6 summarizes these criteria.

The approaches used for comparison were selected from articles published in refereed journals, all with different underlying principles. The common features of these selected articles are that they all consider both direct and indirect trust, and they all deal with Web service composition architectures. The approaches are compared in Table 7 using the criteria stated in Table 6.

The proposed approach does not take into account the filtering of unfair ratings, which could lead to collusion and deception issues. Some academics in the Web service management environment have undertaken research on identifying and filtering unfair ratings [65,66]. However, because there are no differences in their values, distinguishing between fair and unfair ratings is extremely difficult. Any type of customer rating is possible in an open environment because dynamic changes in network performance result in dynamic changes in QoS attributes. The vast majority of system participants are usually trustworthy, according to social network data [67]. The majority of the ratings will most likely be reasonable. Although this assumption is not always valid, it provides the foundation for building a trust/reputation system. A trust/reputation system would be pointless if the bulk of the ratings were unfair.

It is difficult to identify and filter out unfair ratings. Another technique to filter out unfair ratings is to assess the credibility of the raters. A trust evaluation approach should not just filter a rating if it differs from the majority opinion but should also consider whether the rating’s inconsistency is attributable to real-life experience. As a result, just the rater’s credibility is affected, but the rating remains [67]. Customers, for the most part, do not interact directly with one another. The association between trust and customer preference similarity has been proven in some research studies [68,69]. The trust that one customer has in another is based on their similarity in prior preferences. A rating given by a reputable customer carries more weight. This is how the issue is addressed in this work.

It is worth suggesting that the comparison of approaches here does not reflect methodological merits or demerits. Society is large and complicated, trust is full of fuzziness and randomness, these approaches all simulate only a part of trust socialization, and the effectiveness of these approaches can be tested only in the practice of real social relations.

## 7. Conclusions

This work proposes an approach to evaluate Web service trust based on uncertainty methodology. In general, the proposed trust evaluation approach adheres to trust principles and meets the expectations of service customers.

By introducing virtual ratings, this work provides a solution to bootstrapping trust. Even if a service has few or no actual and calculated ratings, it can nevertheless receive an initial trust value and participate in the competition. Furthermore, bootstrapping trust does not require any prior knowledge. Meanwhile, the bootstrapping trust mechanism can reduce decision-making risk by mitigating the negative effects of subjectivity and contingency.

The relationship between a composite service and its component services can be thought of as a whole-to-parts relationship. A composite service is only as trustworthy as its least trustworthy component service. The proposed solution follows this idea, and ensures that both elementary services and composite services are treated without any differences. In other words, the trust evaluation approach can maintain consistency for both elementary services and composite services.

This work integrates the aforementioned solutions in a natural way. The case study demonstrates the viability of the proposed approach.

## Figures and Tables

**Table 1:** An example of service ratings.

Task	Service Class	Service Candidate	# Ratings	Alice’s Rating	Bob’s Rating
$Task_{1}$	$S_{1}$	$s_{1}$	521	0.88 ^1^	0.79
		$s_{2}$	267	0.75	0.90
$Task_{2}$	$S_{2}$	$s_{3}$	0	-	-
		$s_{4}$	244	-	-
		$s_{5}$	125	-	-
$Task_{3}$	$S_{3}$	$s_{6}$	1195	-	-
$Task_{4}$	$S_{4}$	$s_{7}$	3	-	0.83
		$s_{8}$	1540	0.80	-
$Task_{5}$	$S_{5}$	$s_{9}$	935	-	0.81

^1^ Assume that all rating values are real numbers in the interval [0, 1].

**Table 2:** Comparison table of subjective qualitative rating and $t_{l\to i}^{s}$.

Subjective Qualitative Rating	$t_{l\to i}^{s}$
Absolutely Untrustworthy	0
Extremely Untrustworthy	0.1
Very Untrustworthy	0.2
Rather Untrustworthy	0.3
Somewhat Untrustworthy	0.4
Medium	0.5
Somewhat Trustworthy	0.6
Rather Trustworthy	0.7
Very Trustworthy	0.8
Extremely Trustworthy	0.9
Absolutely Trustworthy	1

**Table 3:** Comparison table of the number of actual ratings, the number of calculated ratings and the number of virtual ratings.

Task	Service Candidate	# Actual Ratings ($\eta_{i}$)	# Calculated Ratings ($\gamma_{l\to i}$)	# Virtual Ratings ($\delta_{l\to i}$)
$Task_{2}$	$s_{3}$	0	0	101
	$s_{4}$	244	160.23497	137.44105
	$s_{5}$	125	80.59425	131.4664
$Task_{3}$	$s_{6}$	1195	634.30807	57.856698

**Table 4:** Services comparison after introducing virtual ratings.

Task	Service Candidate	$\nabla_{0.7}\left(T_{l\to i}\right)$	$\nabla_{0.8}\left(T_{l\to i}\right)$
$Task_{2}$	$s_{3}$	0.3069307	0.2079208
	$s_{4}$	0.4628670	0.1680446
	$s_{5}$	0.3217602	0.1776231
$Task_{3}$	$s_{6}$	0.9247469	0.8083127

**Table 5:** Composite services comparison.

Task	Service Candidate	$\nabla_{0.7}\left(T_{l\to i}\right)$	$\nabla_{0.8}\left(T_{l\to i}\right)$
$Task_{C}$	$s_{C}$	0.4280348	0.1358326
	$s_{C^{\prime }}$	0.2838332	0.1680650

**Table 6:** Criteria for the trust approaches in the sector of Web services.

ID	Criterion
C1	Consider the trust dynamism.
C2	Account for both subjective and objective perspectives.
C3	Consider different degrees of ratings.
C4	Consider time decay of ratings.
C5	Filter unfair ratings.
C6	Assess credibility of raters.
C7	Bootstrap trust for the newcomer Web services.
C8	Consider personalized trust evaluation.
C9	Derive trust for composite services.

**Table 7:** Comparison summary between the trust approaches in the sector of Web services.

Approach	C1	C2	C3	C4	C5	C6	C7	C8	C9
Proposed Approach	✔ ^1^	✔	✔	✔	✘ ^2^	✔	✔	✔	✔
Kim et al. [52]	✔	✔	✔	✔	✘	✘	✘	✔	✔
Gao et al. [23]	✔	✔	✔	✘	✘	✘	✔	✔	✔
Karimian et al. [53]	✔	✔	✔	✔	✘	✔	✘	✔	✔

^1^ ✔ represents that the method supports the criterion. ^2^ ✘ represents that the method does not support the criterion.

## Data Availability

Extended Epinions dataset was utilized in this work to highlight the specifics of trust bootstrapping and service composition. The dataset can be found in Trustlet http://www.trustlet.org/extended_epinions.html (accessed on 19 December 2021).
